# DPYD Genotyping, Fluoropyrimidine Dosage and Toxicity: An Umbrella Review of Systematic Reviews

**DOI:** 10.3390/ph18050727

**Published:** 2025-05-15

**Authors:** Sara Otero-Torres, Rosa Rodríguez-Mauriz, Eduard Fort-Casamartina, Ana Clopés-Estela, Francesc Soler-Rotllant, Sandra Fontanals-Martínez, Olalla Montero-Pérez

**Affiliations:** 1 Pharmacy Department, Catalan Institute of Oncology (ICO), Hospital Duran I Reynals, 08098 L’Hospitalet de Llobregat, Spain; rosarmauriz@gmail.com (R.R.-M.); efort@iconcologia.net (E.F.-C.); fsoler@iconcologia.net (F.S.-R.); sfontanals@iconcologia.net (S.F.-M.); olallamontero@iconcologia.net (O.M.-P.); 2 Medicines Department, Catalan Health Service (Catsalut), 08007 Barcelona, Spain; aclopes@catsalut.cat; 3 School of Health Sciences, Blanquerna Ramon Llull University, 08022 Barcelona, Spain; 4 Institut d’Investigació Biomèdica de Bellvitge (IDIBELL), School of Medicine, Universitat de Barcelona (UB), 08908 L’Hospitalet de Llobregat, Spain

**Keywords:** systematic review, DPYD genotyping, fluoropyrimidine, toxicity, clinical oncology

## Abstract

**Background/Objectives**: Fluoropyrimidines are widely used chemotherapeutic agents in various solid tumors. Germline variants in the DPYD gene, which encodes the enzyme dihydropyrimidine dehydrogenase (DPD), are known to impair drug metabolism and increase the risk of severe toxicity. This umbrella review aims to synthesize the current evidence from systematic reviews on the association between DPYD variants and fluoropyrimidine-induced toxicity. **Methods:** A comprehensive search was conducted in PubMed, Web of Science, Scopus, and the Cochrane Library from inception to 2023, including gray literature. Systematic reviews assessing fluoropyrimidine toxicity in oncologic patients with DPYD variants were included. Study quality was assessed using the AMSTAR-2 tool. Registration number in PROSPERO: CRD42023401226. **Results**: Two independent investigators performed the study selection, quality assessment, and data collection. Eight systematic reviews met the inclusion criteria. Methodological confidence was rated as critically low in six, low in one, and medium in another one. The reviews included 125 primary studies, most of them focused on four key *DPYD* variants (DPYD2*A, DPYD*13, c.2846A>T, and HapB3), all of which showed consistent associations with an increased risk of severe toxicity. Rare variants such as DPYD*4, *5, and *6 were also examined, though evidence remains limited. Pharmacogenetics-guided dosing of fluoropyrimidines significantly reduced toxicity rates in several studies. The integration of DPYD genotyping with phenotyping approaches faces limitations; these tests should complement rather than replace genotyping information. **Conclusions**: This umbrella review confirms the clinical relevance of DPYD genotyping to predict and mitigate fluoropyrimidine toxicity. Incorporating genotyping into clinical practice, potentially alongside phenotyping and therapeutic drug monitoring, may enhance patient safety and treatment efficacy.

## 1. Introduction

Fluoropyrimidine-based chemotherapeutic agents, including 5-fluorouracil (5-FU) and its oral prodrugs such as capecitabine, are among the most frequently used treatments for a variety of solid tumors, including colorectal, gastric, breast, and head and neck cancers. These agents function primarily through the inhibition of thymidylate synthase, thereby disrupting DNA synthesis and repair as well as RNA processing.

The enzyme dihydropyrimidine dehydrogenase (DPD), encoded by the DPYD gene, plays a critical role in the initial and rate-limiting step of fluoropyrimidine catabolism. Numerous DPYD polymorphisms have been identified, some of which significantly reduce or eliminate DPD enzymatic activity. While certain variants exert minimal or no clinical impact, others are clearly associated with impaired drug metabolism, resulting in an elevated risk of fluoropyrimidine-induced toxicities, which can range from gastrointestinal side effects to life-threatening complications. Consequently, individuals heterozygous for reduced-function or non-functional DPYD alleles are considered to have partial DPD deficiency. For such patients, dose adjustment of fluoropyrimidine-based regimens is strongly recommended to minimize toxicity and improve treatment safety [1].

Currently, four genetic mutations in the DPYD gene have demonstrated clinically relevant effects on DPD activity in Caucasians, affecting between 3–9% of the population. The frequency of heterozygous genotyping is 1% for *c.1905+1G>A* (also known as DPYD*2A), 0.07–0.1% for *c.1679T>G* (DPYD*13), 1.1% for *c.2846A>T* (p.D949 V), and 2.6–6.3% for *c.1236G>A* (HapB3) [2]. A complete deficiency of DPD activity in carriers of two different variants or the same variant in homozygosity is very rare (0.01–0.50%) [3].

In 2017, the Clinical Pharmacogenetics Implementation Consortium (CPIC) published an updated guideline on DPD genotype and fluoropyrimidine dosing, providing key information on the interpretation of clinical DPYD genotype tests in order to guide clinicians in fluoropyrimidine dose adjustment [4].

Genotyping enables the classification of individuals into three categories: ‘normal’ metabolizers with an activity score of 2, ‘intermediate’ metabolizers with a score between 1 and 1.5, and ‘poor’ metabolizers with a score ranging from 0 to 0.5. While no dose adjustments are necessary for normal metabolizers, intermediate metabolizers should initiate treatment at approximately 50–75% of the standard dose, with the option to increase the dose in subsequent cycles if no toxicity is observed. For poor metabolizers, fluoropyrimidine use is normally contraindicated, and alternative therapeutic strategies should be explored [5].

Phenotypic characterization of DPD deficiency is recommended through the measurement of plasma uracil (U) levels prior to treatment. Elevated pre-treatment U levels are linked to an increased risk of toxicity. While threshold values for complete and partial DPD deficiency remain uncertain, a U level between 16 ng/mL and 150 ng/mL suggests partial DPD deficiency and a higher risk of fluoropyrimidine toxicity. A U level ≥ 150 ng/mL indicates complete DPD deficiency, posing a risk of life-threatening or fatal fluoropyrimidine toxicity [6].

Therapeutic drug monitoring (TDM) of 5-fluorouracil (5-FU) may improve clinical outcomes in patients receiving continuous infusions of 5-FU by reducing toxicities and improving efficacy.

A substantial body of primary research and systematic reviews has explored the association between DPYD gene polymorphisms and adverse reactions to fluoropyrimidine therapy. In 2016, an umbrella review synthesized the existing evidence by evaluating the findings of previously published systematic reviews. This work provided a consolidated analysis of the role of germline DPYD variants in predicting toxicity outcomes, not only in patients treated with fluoropyrimidines but also in those receiving platinum-based chemotherapeutic agents [1].

Since then, numerous systematic reviews and meta-analyses have been conducted, highlighting the growing recognition of DPYD variants beyond the most well-established ones. These more recent studies have examined a broader range of genetic alterations within DPYD, expanding the understanding of their contribution to the risk of severe or potentially life-threatening toxicities associated with fluoropyrimidine treatment. The main objective of this overview of systematic reviews is to identify published systematic reviews on the association between germline variations in the DPYD gene and fluoropyrimidine toxicity. The secondary objective is to assess the association by subgroups, stratified by fluoropyrimidine dosage and cancer type.

## 2. Materials and Methods

The protocol of this systematic review was developed following the Preferred Reported Items for Systematic Review and Meta-analysis Protocols (PRISMA-P) [7] and was registered in the international prospective register of systematic reviews (PROSPERO CRD42023401226) and published in a peer-reviewed journal [8].

The overview of systematic reviews was reported in accordance with the PRISMA statement [7].

### 2.1. Eligibility Criteria

The eligibility criteria for this systematic review were defined according to the PICOS framework (Population, Intervention, Comparison, Outcome, and Study design), as follows:

- P: Oncologic patients with DPYD gene variants and undergoing treatment with fluoropyrimidines.
- I: Registry of severe adverse events (grades 3–5) related to fluoropyrimidine treatment in patients with DPYD gene variants.
- C: Patients without DPYD gene variants and undergoing treatment with fluoropyrimidines or without comparator.
- O: Variables related to toxicity and treatment: severe adverse events, DPYD gene variants detected, fluoropyrimidine dosage, and treatment regimen.
- S: Systematic review with/without meta-analysis.

The exclusion criteria encompassed reviews that did not adhere to a systematic review methodology, studies conducted in vitro or in animal models, and those in which genotype data could not be extracted or the relevant information was insufficient.

Moreover, no restrictions were applied regarding the publication date or language.

### 2.2. Information Sources and Search Strategy

A comprehensive search was conducted, covering all available articles from inception until February 2023 in four healthcare peer-reviewed databases: PubMed, Web of Science, Scopus (Elsevier Science), and the Cochrane Library. A combination of Medical Subject Headings (MeSH) and free-text terms combined with Boolean operators was used as displayed in Table S1 (Supplementary Materials).

Gray literature was gathered through searches in Google Scholar as well as the reference lists of identified relevant articles. Common registry databases such as TESEO and the PROSPERO Register were also searched.

### 2.3. Selection and Data Collection Process

A peer-review of the literature was performed by two independent investigators (S.O.-T. and R.R.-M.), who screened the titles and abstracts of all potential systematic reviews for possible inclusion, with any discrepancy settled by consensus or with a third reviewer (O.M.-P.). Two reviewers (S.O.-T. and R.R.-M.) then independently extracted data from the included systematic reviews, and each examined the extraction sheet of the other in order to ensure accuracy and reach consensus. Inter-rater agreement was calculated with the kappa coefficient using Stata Statistical Software version no. 18.

If there were any data missing from a review, it was explicitly stated. Table 1 summarizes the variables registered for each systematic review.

### 2.4. Quality Assessment

One reviewer (O.M.-P.) carried out the assessment of the quality of the systematic reviews using a critical appraisal tool designed for this purpose, namely A Measurement Tool to Assess Systematic Reviews 2 (AMSTAR 2) [9]. This tool is not intended to generate an overall score, but rather to highlight relevant items and their potential impact on the overall confidence of the systematic review.

According to the tool, the overall confidence can be rated as high, moderate, low, and critically low.

## 3. Results

The electronic search resulted in 79 publications found across the databases reviewed, with one additional publication identified through a search of gray literature. Out of the 80 total publications, 20 were removed due to duplication.

**Figure 1:**
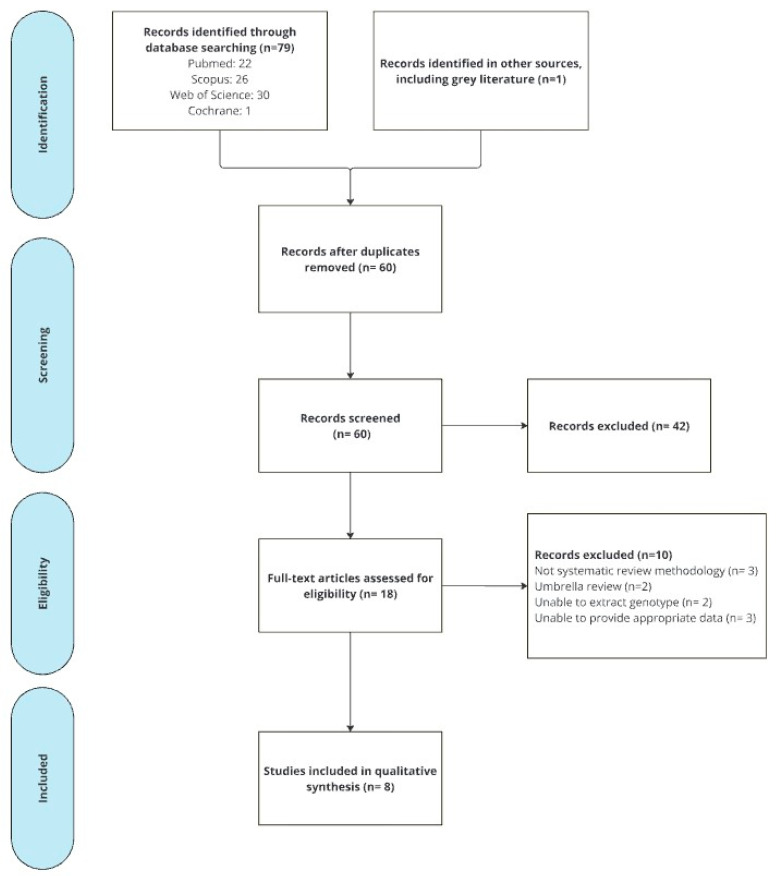
Preferred reporting items for systematic reviews and meta-analyses literature search and study selection flowchart.

Inclusion and exclusion criteria were applied to titles and abstracts by two investigators with 91.5% agreement (kappa 0.8 standard error 0.13), resulting in 42 publications excluded. This left 18 potentially relevant reviews, which were retrieved in full text for further evaluation. Ten of these were excluded (Table S2, Supplementary Materials), and eight fulfilled the inclusion criteria (refer to Figure 1).

### 3.1. Quality of the Systematic Reviews

Table S3 (Supplementary Materials) reports the results for each domain of the AMSTAR-2 tool [9]. The overall quality of the included systematic reviews was poor. Of the eight reviews, six of them were rated as critically low confidence and one of them as low confidence. Only one was rated as medium.

All the included systematic reviews have weaknesses in different items of the AMSTAR-2 tool [9], ranging between three and six in most of them, with the exception of the reviews of Rosmarin et al. [10] and Conti et al. [11], which present nine compromised items.

Regarding critical domains, all the reviews have at least one critical flaw, with the exception of Ontario Health [12].

Six reviews do not contain an explicit statement indicating that the review methods were established in a protocol prior to conducting the review. Only the reviews of Ontario Health [12] and Glewis et al. [13] included this statement. Additionally, six reviews did not provide a list of excluded studies assessed via a reading of the full text and justifying the reason for their exclusion. Only the reviews of Ontario Health [12] and Paulsen et al. [14] provided such lists.

These two items were the most frequently affected among the critical domains. Furthermore, Rosmarin et al. [10], Conti et al. [11], and Paulsen et al. [14] did not use a satisfactory technique for assessing the risk of bias in individual studies, nor did they account for the risk of bias in individual studies when interpreting/discussing the results. Rosmarin et al. [10] did not use a comprehensive literature search strategy according to the AMSTAR-2 criteria either [9].

Regarding non-critical domains, all the reviews have at least two affected items.

None of the reviews reported on the sources of funding for the studies included, and only half of the authors performed duplicate study selection. The same proportion performed duplicate data extraction.

### 3.2. Characteristics of Included Systematic Reviews

Full details of the studies included are shown in Table 2. All of the included systematic reviews aim to assess the association between the risk of severe fluoropyrimidine-related toxicity and the presence of DPYD gene variants. The reviews included a variable number of primary studies, ranging from six to twenty-nine studies.

In terms of tumor type, colorectal cancer was found to be the predominant tumor type in most systematic reviews, although other cancers such as breast, gastric, esophageal, biliary, pancreatic, and head and neck cancers were also represented. In the reviews by Paulsen et al. [14], Rosmarin et al. [10], and Conti et al. [11], the specific types of tumors treated with fluoropyrimidine-based therapies were not explicitly specified.

Regarding the DPYD variants, four reviews [11,12,14,15] included all four DPYD variants under evaluation (DPYD*2A, DPYD*13, *c.2846A>T*, and HapB3). One study [10] evaluated three variants (DPYD 2A, *c.2846A>T*, HapB3), one study [16] focused on two variants (DPYD 2A, *c.2846A>T*), and one study [13] assessed a single variant (DPYD 2A). Other rare variants were studied in several investigations: DPYD *6 [10,11,17], *c.1601G>A* [10,15], DPYD *496A>G*, and DPYD*5 *1627A>G* and DPYD *85T>C* [10].

In terms of chemotherapy regimens, most studies reported 5-FU as the predominant drug, either alone or in combination. Capecitabine was also frequently used.

The reviews reported results concerning fluoropyrimidine severe toxicity (grade 3–5) using the National Cancer Institute Common Toxicity Criteria (NCI–CTC) [18], with the exception of two studies that used the WHO criteria [16]. Two reviews provided qualitative results [11,14], five contributed quantitative results in the form of meta-analyses [10,13,15,16,17], and one study provided both qualitative and quantitative results with no heterogeneity calculated [12].

Three studies received no funding. The study by Kim et al. [17] was funded by the Korean government. The review by Conti et al. [11] was funded by the Italian Medicines Agency (AIFA) AVPM/17806/A (Rome, Italy) and Reti Oncologiche, Campania Region (2018). The review by Ontario Health [12] received funding from Ontario Health. The study by Paulsen et al. [14] received grants from the Danish Cancer Society and the Region of Southern Denmark. Funding information is not available for the review by Rosmarin et al. [10].

### 3.3. Fluoropyrimidine-Induced Toxicity DPYD

#### 3.3.1. Germline Variations in the DPYD Gene and Fluoropyrimidine Toxicity (Table 3 and Table 4)

The included reviews examine the relationship between the presence of DPYD gene variants and the occurrence of severe adverse reactions associated with the administration of fluoropyrimidines. The results are grouped according to the specific variant altered, although one review reported results on the toxicity of any of the four DPYD variants.

The Ontario Health review [12] gave qualitative results according to each of the specific variants altered but also pooled the results of severe toxicity depending on whether any or no altered variants were present. Overall toxicity ranged from 23.5% to 100.0% in DPYD variant carriers versus 8.2% to 41.5% in WT patients in the pooling results of seven studies, with a risk ratio (RR) of 2.6 (95% confidence interval [CI] 2.2–4.0).

Notably, the incidence of diarrhea and neutropenia was significantly higher among DPYD variant carriers than in wild type (WT) patients. Neutropenia occurred more frequently in DPYD carriers (up to 35.3%) compared to 6.5% in WT patients, with a pooled RR of 4.4 (95% CI 1.6–9.2).

- Carriers of *c.1905+1G>A* (*rs3918290*) [also known as DPYD*2A] variant

Five reviews [10,11,12,15,16] evaluated the association between DPYD*2A and toxicity.

Two reviews [15,16] showed statistical associations between overall toxicity and DPYD*2A. Terrazzino et al. [16] observed an Odds Ratio (OR) of 5.4 (95% CI 2.8–10.5, *p* < 0.001), with no significant heterogeneity observed among studies (*p* = 0.3; I^2^:13%). Additionally, they identified a significantly increased risk of hematological toxicity (OR 15.8, 95% CI 6.4–39.1, *p* < 0.001), diarrhea (OR 5.5, 95% CI 2.3–13.3, *p* < 0.001), and mucositis (OR 7.5, 95% CI 3.0–18.5, *p* < 0.001), without heterogeneity among studies (I^2^ = 0%). Similar results were reported in the review by Meulendijks et al. [15], with an overall toxicity of adjusted RR 2.9 (95% CI 1.8–4.6, *p* < 0.0001) but a high associated heterogeneity (I^2^ = 73%, *p* = 0.0013).

Both the reviews, Conti et al. [11] and Ontario Health [12], did not perform quantitative analyses. In the review by Conti et al. [11], only four studies evaluated the association between the DPYD*2A variant and fluoropyrimidine-related toxicity. Three of the included studies confirmed the association. However, one study did not find a significant association between DPYD*2A and toxicity. In the Ontario Health review [12], 16 out of 18 studies reported overall toxicity ranging from 46.2% to 100% in DPYD*2A carriers versus 3.3% to 57.5% in WT patients. Neutropenia occurred in 33% to 100% of DPYD*2A carriers compared to 2% to 36% of WT patients across nine studies. Diarrhea was more common in DPYD*2A carriers (12.0% to 100.0%) compared to 1.4% to 27.5% in WT patients across nine studies.

Finally, Rosmarin et al. [10] reviewed the association of the DPYD*2A polymorphism with capecitabine and 5-FU global toxicity, showing a non-significant association for capecitabine (OR 3.0, 95% CI 0.8–11.7, *p* = 0.1) without heterogeneity (*p* = 0.8), but a statistically significant association for infusional 5-FU (OR 6.7, 95% CI 1.7–27.1, *p* = 0.0075). However, in 5-FU bolus treatment, DPYD2A did not reach statistical significance for global toxicity (OR 0.7, 95% CI 0.5–1.0, *p* = 0.062).

Regarding gastrointestinal toxicity, no significant association was found for capecitabine (OR 3.1, 95% CI: 0.71–13.9, *p* = 0.1), or for 5-FU bolus (OR 1.47, 95% CI: 0.2–12.2, *p* = 0.7). In contrast, an association with diarrhea was statistically significant for infusional 5-FU (OR 7.7, 95% CI: 1.6–36.9, *p* = 0.011).

With respect to hematological toxicity, the 5-FU bolus was significantly associated with neutropenia (OR 12.9, 95% CI 3.1–53.3, *p* = 0.0004), but it was not evaluated for capecitabine or infusional 5-FU.

- Carriers of *c.1679T>G* (*rs55886062*) [also known as DPYD*13] variant

The DPYD*13 variant was evaluated in three systematic reviews [11,12,15].

The review of Meulendijks et al. [15] found a significant association between *c.1679T>G* and overall toxicity, with an adjusted RR of 4.4 (95% CI 2.1–9.3, *p* < 0.0001), and high association with heterogeneity (I^2^ = 85%, *p* < 0.0001). This variant was particularly associated with hematological toxicity (adjusted RR 9.8, 95% CI 3.0–31.5, *p* = 0.00014) and gastrointestinal toxicity (adjusted RR 5.7, 95% CI 1.4–23.3, *p* = 0.015).

The reviews of Conti et al. [11] and Ontario Health [12] did not perform quantitative analyses. In the review by Ontario Health, five out of seven studies reported positive results on the association between *c.1679T>G* and overall toxicity, ranging from 50% to 100% in DPYD*13 carriers compared to 8.2% to 49.5% in WT patients. The results were also significant in the three studies that reported diarrhea, ranging from 50% to 100% of DPYD*13 carriers compared to 5.8% to 22% of WT patients. Regarding neutropenia, two studies evaluated its association with the *c.1679T>G* variant, but no consistent association was found.

In the review by Conti et al. [11] one study detected a correlation between the DPYD*13 variant and adverse events, although it did not achieve statistical significance.

- Carriers of *c.2846A>T* (*rs67376798*) variant

The *c.2846A>T* variant was analyzed in four reviews [11,12,15,16]. Two of them [15,16] showed a significant association between this variant and severe fluoropyrimidine-related toxicity.

In the review by Meulendijks et al. [15], the *c.2846A>T* variant was significantly associated with severe toxicity. The adjusted RR for this association was 3.0 (95% CI 2.2–4.1, *p* < 0.0001), although high heterogeneity was observed (I^2^ = 80%, *p* < 0.0001), suggesting variability among the included studies.

Similarly, the review by Terrazzino et al. [16] reported an increased risk of overall toxicity (OR 8.2 95% CI 2.7–25.3, *p* < 0.001). For diarrhea, the pooled OR was 6.0 (95% CI 1.8–20.7, *p* = 0.004). While moderate heterogeneity was observed for overall toxicity (I^2^ = 47%, *p* = 0.076), no heterogeneity was reported for diarrhea (I^2^ = 0%).

In the review by Conti et al. [11], four studies evaluated the *c.2846A>T* variant, three of them reported a significant association between this variant and fluoropyrimidine-associated toxicity, although one study did not detect the variant in their patient population.

Lastly, the review by Ontario Health [12] evaluated the *c.2846A>T* variant in 13 studies. One of them found no severe toxicity in *c.2846A>T* carriers treated with standard fluoropyrimidine doses, while 12 studies showed a carrier frequency of severe toxicity ranging from 60% to 100%, compared to 3.3% to 50.1% in WT patients.

- Carriers of *c.1236G>A* (*rs75017182*) [also known as HapB3] variant

The *c.1236G>A* [HapB3] variant has been analyzed in two reviews, both showing statistically significant results [12,15].

In the review by Meulendijks et al. [15], a significant association was observed between HapB3 and overall toxicity, with an adjusted RR of 1.6 (95% CI 1.3–2.0, *p* < 0.0001). Heterogeneity across studies was low (I^2^ = 23%, *p* = 0.26). This variant was most strongly associated with gastrointestinal toxicity (adjusted RR 2.04 95% CI 1.5–2.8, *p* < 0.0001) and hematological toxicity (adjusted RR 2.1, 95% CI 1.2–3.7, *p* = 0.013).

Lastly, Ontario Health [12] evaluated the *c.1236G>A* variant in nine studies. Six of them reported that overall toxicity ranged from 30% to 92.9% in heterozygous *c.1236G>A* carriers versus 8.2% to 85% in WT patients. Regarding neutropenia, one study evaluated its association with the *c.1236G>A* variant with a frequency of severe toxicity of 22.1% compared to 9.8% in WT patients. Diarrhea was measured in two studies that showed a carrier frequency of diarrhea ranging from 14.3% to 50%, compared to 12.5% to 23.1% in WT patients.

- Carriers of other rare variants vs. WT patients
  o Carriers of *c.1601G>A* (*rs1801158*) [DPYD*4] variant

The DPYD*4 variant was explored in the review by Meulendijks et al. [15], with no significant association between *c.1601G>A* and severe fluoropyrimidine-associated toxicity. The adjusted RR was 1.5 (95% CI 0.9–2.7, *p* = 0.15), with a high association with heterogeneity (I^2^ = 91%, *p* < 0.0001). A strong association was observed between c.1601G>A and severe gastrointestinal toxicity (RR 2.0, 95% CI 1.5–2.8, *p* < 0.0001) and hematological toxicity (RR 1.9, 95% CI 1.2–3.3, *p* = 0.12).

 o Carriers of *c.2194G>A* (*rs1801160*) [DPYD*6] variant

DPYD*6 was explored in two of the included reviews [11,17].

Kim et al. [17] showed that *rs1801160* polymorphism was significantly associated with an increased risk of overall toxicity (OR 1.7, 95% CI 1.4–2.1, *p* < 0.001); and moderate heterogeneity (I^2^ = 30%, *p* = 0.21).

In the review by Conti et al. [11], three studies measured the DPYD*6 variant without pulling the results, with only one study reporting association between the DPYD*6 variants and the occurrence of severe neutropenia (OR 2.6, 95% CI 1.4–5.0, *p* = 0.0041).

 o Carriers of *c.496A>G* (*rs2297595*) variant

Rosmarin et al. [10] explored the association between *c.496A>G* and global toxicity, without significant results in either bolus 5-FU treatment (OR 1.3 95% CI 0.8–2.0, *p* = 0.35) or infusional 5-FU (OR 0.5, 95% CI 0.1–3.1, *p* = 0.48). Regarding diarrhea, no significant association was observed with either bolus 5-FU (OR 1.4, 95% CI 0.8–2.4, *p* = 0.22) or infusional 5-FU (OR 0.4, 95% CI 0.0–3.4, *p* = 0.38). Regarding neutropenia, no significant association was observed with bolus 5-FU (OR 1.2, 95% CI 0.52–2.60, *p* = 0.70).

 o Carriers of *c.1627A>G* (*rs1801159*) [DPYD*5] variant

Rosmarin et al. [10] examined the association between 5-FU treatment and overall toxicity in patients carrying the DPYD*5 variant, which did not reach statistical significance in bolus (OR 0.7, 95% CI 0.5–1.0, *p* = 0.062) or infusional 5-FU (OR 0.68, 95% CI 0.3–1.8, *p* = 0.43).

Regarding diarrhea, no significant association was observed with either bolus 5-FU (OR 0.8, 95% CI 0.5–1.3, *p* = 0.34) or infusional 5-FU (OR 0.7, 95% CI 0.2–2.1, *p* = 0.48). For neutropenia, no significant association was observed with bolus 5-FU (OR 0.7, 95% CI 0.4–1.5, *p* = 0.39).

**Table 3:** Pooled results of severe overall toxicity of the main variants.

Review	DPYD*2A			DPYD*13			c.2846A>T			HapB3		
	Risk (95% CI)	*p*	Heterogeneity	Risk (95% CI)	*p*	Heterogeneity	Risk (95% CI)	*p*	Heterogeneity	Risk (95% CI)	*p*	Heterogeneity
Meulendijks et al. (2015) [15]	RR 2.9 (1.8–4.6)	*p* < 0.0001	I^2^ = 73% *p* = 0.0013	RR 4.4 (2.1–9.3)	*p* < 0.0001	I^2^ = 85% *p* < 0.0001	RR 3.0 (2.2–4.1)	*p* < 0.0001	I^2^ = 80% *p* < 0.0001	RR 1.6 (1.3–2.0)	*p* < 0.0001	I^2^ = 23%, *p* = 0.26
Terrazzino et al. (2013) [16]	OR 5.4 (2.8–10.5)	*p* < 0.001	I^2^ = 13% *p* = 0.3	-			OR 8.2 (2.7–25.3)	*p* < 0.001	I^2^ = 47% *p* = 0.076	-		
Rosmarin et al. (2014) [10]	Capecitabine: OR 3.0 (0.8–11.7)	*p* = 0.1	I^2^ = 0% *p* > 0.05	-			-			-		
	Infusional 5-FU: OR 6.7 (1.7–27.1)	*p* = 0.0075	NR									
	Bolus 5-FU: OR 0.7 (0.5–1.0)	*p* = 0.062	NR									

**Table 4:** Pooled results of severe overall toxicity of rare variants.

Review	DPYD*4			DPYD*6			c.496A>G			DPYD*5		
	Risk (95% CI)	*p*	Heterogeneity	Risk (95% CI)	*p*	Heterogeneity	Risk (95% CI)	*p*	Heterogeneity	Risk (95% CI)	*p*	Heterogeneity
Meulendijks et al. (2015) [15]	RR 1.5 (0.9–2.7)	*p* = 0.15	I^2^ = 91%, *p* < 0.0001									
Rosmarin et al. (2014) [10]	-			-			Infusional 5-FU: OR 0.5 (0.1–3.1)	*p* = 0.48	NR	Infusional 5-FU: OR 0.68 (0.3–1.8)	*p* = 0.43	NR
							Bolus 5-FU: OR 1.3 (0.8–2.0)	*p* = 0.35	NR	Bolus 5-FU: OR 0.7 (0.5–1.0)	*p* = 0.062	NR
Kim et al. (2022) [17]	-			OR 1.7 (1.4–2.1)	*p* < 0.001	I^2^ = 30% *p* = 0.21	-			-		

#### 3.3.2. Fluoropyrimidine Pharmacogenetics-Guided Dosing and Toxicity

Pharmacogenetics-guided dosing (PGD) is a strategy aimed at reducing toxicity by adjusting fluopyrimidine doses before starting the treatment based on the study of DPYD gene variants. Three of the reviews examined the role of DPYD genotyping in guiding fluoropyrimidines dosing to minimize severe adverse effects [12,13,14].

The review by Glewis et al. [13] compared PGD cohorts to non-PGD cohorts and showed that PGD significantly reduced the risk of overall toxicity (RR 0.3, 95% CI 0.3–0.4; *p* < 0.00001) with low associations with heterogeneity (I^2^ = 32%, *p* = 0.21). Additionally, PGD cohorts exhibited a reduced risk of diarrhea (RR 0.4, 95% CI 0.2–0.6, *p* < 0.0001), without heterogeneity (I^2^ = 0%, *p* = 0.92).

Regarding the PGD cohort, when comparing carriers of DPYD variants with WT patients, three out of five studies reported higher incidences of specific toxicities such as diarrhea, nausea, vomiting, and hand-foot syndrome in WT patients. However, three out of five studies found that DPYD variant carriers experienced higher overall toxicity and gastrointestinal issues.

The review by Paulsen et al. [14] included 12 studies with non-PGD cohorts in which the prevalence of severe toxicity varied significantly. In WT patients, the prevalence of overall toxicity ranged from 10% to 49%, while in patients with DPYD variants, it ranged from 14% to 89%. In contrast, three studies investigating PGD cohorts yielded mixed findings. Wigle et al. [19] observed reduced toxicity rates among DPYD variant carriers who received pre-treatment dose adjustments. However, Lunenburg et al. [20] reported that DPYD carriers receiving dose reductions experienced toxicity rates similar to those who received standard doses, while Henricks et al. [21] found higher toxicity rates in the dose-reduced group compared to the WT cohort.

Six studies by Ontario Health et al. [12] evaluated the risk of severe toxicity in DPYD carriers treated with a PGD-reduced fluoropyrimidine dose versus the risk in WT patients. Severe toxicity occurred in 18% to 50% of DPYD carriers with a reduced dose, compared to 14% to 38% of WT patients. Only Henricks et al. [21] reported a higher risk of severe hematological and gastrointestinal toxicity in DPYD carriers treated with a reduced dose, while other studies yielded inconclusive results. In Lunenburg et al. [20], 9.1% of DPYD carriers on a reduced dose experienced severe hematological and gastrointestinal toxicity, compared to 11.8% of those on a standard dose with severe hematological toxicity and 17.6% with severe gastrointestinal toxicity.

### 3.4. Combined Genotyping and Phenotyping Approaches and Toxicity

In two of the selected reviews, the combination of genotype and phenotype methods was explored [11,14]. While genotyping identifies genetic predispositions, phenotyping assesses the actual DPD enzyme activity. Thus, measuring the plasma levels of uracil (U) and/or its metabolite dihydrouracil (UH2)—indicative of fluorouracil clearance—holds promise as a diagnostic approach, though it has not yet been incorporated into routine clinical use.

In the review by Paulsen et al. [14], three studies evaluated DPD phenotype and the four clinically relevant DPYD variants, but they did not link these findings to toxicity. The study published by De With et al. [22] found that the median level of [U] differed between patients with DPYD variants and WT patients (WT: 10.1 ng/mL, HapB3: 12.2, *c.2846A > T*: 14.6, DPYD*2A: 16.8, DPYD*13: 40.1 ng/mL). In contrast, Etienne-Grimaldi et al. [23] observed that only c.2846A>T was associated with elevated [U]. Capitain et al. [24] did not provide information on [U] levels according to specific DPYD variants.

Ten studies included in the review of Conti et al. [11] integrated DPYD genotyping with phenotyping methods with reported results. Six of them indicated a correlation between genotype and phenotype. Among them, Van Kuilenburg et al. [25] suggested that patients with reduced DPD enzymatic activity experienced faster onset and more severe toxicity. Similarly, Henricks et al. [21] analyzed four recommended DPYD variants and performed phenotyping tests. They confirmed that carriers of DPYD *c.1236G>A* and *c.2846A>T* are more prone to severe FP-related toxicity. In addition, the mean DPD enzyme activity was significantly lower in patients bearing these two genetic variants, as well as DPYD*2A, compared to other patients. Only one patient carrying DPYD*13 showed a 60% DPD activity reduction. This patient was treated with a reduced 5-FU dosage for three treatment cycles, and no severe toxicity occurred.

Conversely, four studies were unable to establish this correlation due to various limitations, such as small sample sizes or the absence of detected DPYD variant carriers in their cohorts. Among them, Boisdron et al. [26] conducted a study combining pharmacogenetic-guided dosing with UH2/U ratio measurements and observed a low incidence of severe adverse events, even with significant dose increases in patients who used a dose guiding approach. However, the small sample size again limited conclusions about whether phenotyping enhanced the predictive power of DPYD genotyping.

## 4. Discussion

This umbrella review provides an updated synthesis of the relationship between germline DPYD gene variants and fluoropyrimidine-induced severe toxicity. By consolidating and updating findings from recent systematic reviews, our study enhances the understanding of DPYD pharmacogenetics and its role in optimizing fluoropyrimidine therapy.

The umbrella review published in 2016 by Campbell et al. [1] synthesized several systematic reviews examining DPYD polymorphisms and fluoropyrimidine toxicity. They found that the DPYD*2A variant was strongly associated with increased global toxicity, particularly severe diarrhea, hematological toxicity, and mucositis. The c.2846A>T variant also showed a consistent association with an elevated toxicity risk, establishing DPYD alterations as the most clinically relevant predictors of fluoropyrimidine toxicity compared to other genes like TYMS or MTHFR.

Building upon this previous work, the present review integrates more recent findings that reinforce the critical role of DPYD genotyping in clinical practice. The CPIC [4], Dutch Pharmacogenetics Working Group (DPWG) [27], Réseau National de Pharmacogénétique (RNPGx) [28], Sociedad Española de Farmacogenética y Farmacogenómica (SEFF), and the Sociedad Española de Oncología Médica (SEOM) [5] clinical guidelines all recommend genotyping the four main DPYD variants that are most strongly associated with fluoropyrimidine toxicity.

The recommendations from the SEFF [29] identify several additional rare variants of potential clinical relevance, such as *rs115232898* (*c.557A>G*), *rs1801266* (DPYD*8, *c.703C>T*), *rs1801268* (DPYD*10, *c.2983G>T*), *rs72549309* (DPYD*7, *c.295_298delTCAT*), *rs78060119* (DPYD*12, *c.1156G>T*), and *rs72549303* (DPYD*3, *c.1898delC*). Although these variants are rare in the population (Minor Allele Frequency <0.001), they have strong or moderate evidence supporting reduced function or a complete loss of function, contributing to the variability in DPD enzymatic activity.

In the presented work, two investigators independently conducted a systematic review of the literature with minimal restrictions, including all systematic reviews focusing on the study of patients with genetic study of DPYD gene variants under treatment with fluoropyrimidines and related toxicity. The goal was to identify as many systematic reviews as possible and group the evidence available to date.

The confidence of the systematic reviews included is critically low, except for the review by Glewis et al. [13], which is low, and the review by Ontario Health [12], which is of moderate confidence according to the AMSTAR 2 tool criteria [9]. Each of the reviews had deficiencies in different items of the checklist; however, the lack of a list of excluded studies and not reporting an explicit statement that a registered review protocol exists prior to the start of the review were the most frequent in terms of critical items.

Only Ontario Health [12] and Glewis et al. [13] reported the existence of a protocol. Adherence to a well-developed protocol reduces the risk of bias in the review. Second, only Ontario Health [12] and Paulsen et al. [14] provided a list of excluded studies with the reasons for their exclusion, which is critical to assess the risk of bias that may be implied in the results by the unjustified exclusion of studies.

As to non-critical items, none of the reviews reported on the sources of funding for the studies included, which is essential to guarantee transparency and can sometimes be relevant to making subgroup comparisons if the source of funding is related to the intervention of the study.

The four key DPYD variants currently included in clinical guidelines were also shown to be associated with severe fluoropyrimidine-related toxicity in this systematic review. Compared to the previous umbrella review [1], our overview of systematic reviews expands on earlier findings by including additional clinically relevant DPYD variants such as DPYD*13 and HapB3. Beyond the four well-established DPYD variants, this review also highlights the growing evaluation of other rare variants, such as DPYD*4, DPYD*5, DPYD*6, and c.496A>G. While the systematic reviews included in this umbrella review explore a different set of less common variants than that addressed in the SEFF recommendations [29], this diversity in evidence and research focus underscores the need for expanded genotyping panels to capture a broader spectrum of DPD deficiencies while also recognizing the variability and limitations of the current evidence [4,5,27,28].

Importantly, several reviews included in this umbrella review provide updated evidence regarding fluoropyrimidine dose adjustments based on DPYD genotyping as well as toxicity associations stratified by fluoropyrimidine pharmacogenetics-guided dosing. Dose reduction strategies have consistently demonstrated a significant decrease in the incidence of severe toxicity. These findings reflect advancements beyond the previous umbrella review; advancements that align with current clinical guidelines from CPIC [4], DPWG [27], RNPGx [28], and SEFF/SEOM [5].

The present study emphasizes the integration of DPYD genotyping with phenotyping approaches. However, these phenotypic methods face limitations, including limited accessibility and inconsistent correlations between DPD activity and fluoropyrimidine toxicity. This umbrella review supports genotyping as the preferred approach, as endorsed by SEFF recommendations [29], with clinical validation needed before phenotypic tests can be effectively implemented in practice. At present, these tests should complement rather than replace genotyping information.

In line with these recommendations, the SEFF [29] also highlights the value of TDM of 5-FU, using specific AUC and plasma concentration targets for different cancer regimens to improve treatment response and reduce adverse events. However, TDM should not be used as a substitute for pre-treatment DPYD genotyping, as it may be ineffective in preventing severe toxicity in intermediate or poor metabolizers.

Nonetheless, this review is not without limitations. The main one is the low quality of the studies included, which weakens the strength of the conclusions. Another limitation to be taken into account is the heterogeneity observed in some of the included studies, particularly reflected by high I^2^ values in key associations such as *DPYD*2A and *DPYD*13. This variability likely stems from differences in the study populations, dosing regimens, and outcomes definitions across the included studies. Additionally, most of the systematic reviews included predominantly Caucasian cohorts, limiting the applicability of the results to other populations. Furthermore, the low prevalence of DPYD variants makes it difficult to gather large enough samples for statistically significant results, especially for rare variants, which limits the generalizability of the findings.

Future research should prioritize expanding genetic panels to include additional rare and novel DPYD variants or the use of DPYD full-gene sequencing in large patient cohorts, improving sensitivity for toxicity prediction. The validation of these rare variants and their integration into routine clinical practice is essential to enhance patient care. Upcoming studies should include more ethnically diverse cohorts in order to determine whether these findings hold true in underrepresented populations.

Since not all toxicity is DPYD-related, research might expand to other genetic factors that, in combination with DPYD, could better predict fluoropyrimidine tolerance. Additionally, validating the integration of genotyping, phenotyping, and TDM of 5-FU in large multicenter studies will refine individualized treatment strategies. Finally, evidence-based clinical guidelines should incorporate these tools to ensure safe and effective routine oncology care.

## 5. Conclusions

This umbrella review confirms a consistent and clinically meaningful association between the four principal DPYD variants and the occurrence of severe fluoropyrimidine toxicity across multiple tumor types. Although the data on rarer alleles remain preliminary, emerging signals suggest that they too may contribute to heightened toxicity risk in affected patients.

Importantly, studies of reducing the initial fluoropyrimidine dose in DPYD variant carriers consistently demonstrate substantial reductions in life-threatening adverse events compared with standard body-surface-area dosing. These results underscore the immediate benefit of incorporating DPYD genotyping into routine oncology practice to identify at-risk individuals and tailor therapy before treatment initiation.

Beyond genotyping alone, integrating DPYD results with DPD phenotyping and 5-FU TDM represents a multifaceted approach to further refine dose selection and enhance patient safety, particularly for intermediate and poor metabolizers. While phenotypic and pharmacokinetic tools should complement rather than replace genotyping, their combined use holds promise for fully personalized fluoropyrimidine regimens.

Future large-scale studies should validate expanded variant panels and optimized algorithms, ensuring that comprehensive pharmacogenetic screening and dose-adjustment protocols are embedded within clinical guidelines to maximize both the efficacy and the safety of fluoropyrimidine chemotherapy.

## Figures and Tables

**Table 1:** Variables collected.

General Variables
Author and year of publication
Aim of systematic review
Number of primary studies
Design of primary studies
Number of participants/Caucasians
Tumor type
Funding statement
Competing interest statement
**Specific Variables**
Severe adverse events (overall toxicity, gastrointestinal toxicity, hematological toxicity)
DPYD gene variants detected
Fluoropyrimidine guided dosing
Chemotherapeutic regimen

**Table 2:** Main characteristics of the systematic reviews included.

Author/Year	Aim	Primary Studies (n)	Primary Studies Design	Participants (n)/Caucasians (%)	Tumor Type	DPYD Genotype	Chemotherapeutic Regimens	Toxicity Criteria
Meulendijks et al. (2015) [15]	To assess the clinical relevance of DPYD*13, HapB3, and DPYD*4 as predictors of severe FIT.	8	Cohort studies and RCTs	7365/85–100%	Colorectal, Gastric/gastroesophageal, hepatobiliary and pancreatic, breast and others.	DPYD*13: 5 studies (5616 patients) HapB3: 6 studies (4261 patients) DPYD*2A: 7 studies (5737 patients) c.2846A>T: 8 studies (7318 patients). DPYD*4: 5 studies (3900 patients)	Capecitabine: 2 studies 5-FU: 2 studies capecitabine and 5-FU regimens: 4 studies	NCI–CTC
Terrazzino et al. (2013) [16]	To quantify the impact of the DPYD*2A and 2846A>T variants on the risk of FIT, to determine sensitivity, and specificity testing for DPYD variants.	15	Prospective and retrospective studies	4573/NR (mostly Caucasians)	Colorectal: predominant. Others: GI, head and neck and breast cancers.	DPYD*2A: 13 studies (3499 patients) c.2846A>T: 7 studies (2308 patients).	Capecitabine: 2 studies Tefagur-uracil: 1 study. In the remaining studies: 5-FU or capecitabine.	NCI–CTC: 13 studies WHO criteria: 2 studies
Kim et al. (2022) [17]	To investigate the association between DPYD*6 and FIT.	6	RCTs and cohort studies.	6119/100%	Colorectal, breast, biliary, pancreatic, orofacial, esophageal, and gastric cancers.	DPYD*6	Fluoropyrimidine-based regimens: 4 studies FOLFOX4: 1 study Capecitabine: 1 study	NCI–CTC
Conti et al. (2020) [11]	To analyze the variability of responses to fluoropyrimidine-based chemotherapy by DPYD genotyping combined with phenotyping methods and/or clinical monitoring.	22	Observational and RCTs.	18,018/NR	NR	DPYD*13, HapB3, DPYD*2A, c.2846A>T and DPYD*6	5-FU or capecitabine.	NCI–CTC
Rosmarin et al. (2014) [10]	To investigate the associations between fluoropyrimidine- polymorphisms and FIT.	16	RCTs and cohort studies.	4855/100%	NR	DPYD*9A, c.496A>G, HapB3, DPYD*4, DPYD*5, DPYD*2A, DPYD*6, and c.2846A>T	Bolus and infusional 5-FU or capecitabine.	NCI–CTC
Glewis et al. (2022) [13]	To evaluate treatment outcomes between PGD versus non-PGD and within PGD	17	Cohort studies and case-control study	11,515/NR (mostly Caucasians)	Lower GI); upper GI; breast cancer; head and neck cancers); and gynecological cancers	Studies with majority testing for The bold formatting is not necessary, so we will remove it. 4o (15 studies)	5-FU: 14 studies. Capecitabine: 11 studies	NCI–CTC
Ontario Health (2021) [12]	To evaluate the risk of severe FIT in carriers of the DPYD variants compared to patients with wild-type DPYD.	29	Observational studies,	18,490/67–100%	Colorectal: predominant Other: Breast, GI, esophageal, and head and neck.	Four DPYD variants: 4 studies DPYD*2A: 20 studies c.2846A>T: 16 studies DPYD*13: 13 studies	5-FU: 11 studies. Capecitabine: 4 studies. In the remaining studies: 12–91% of patients with 5-FU.	NCI–CTC
Paulsen et al. (2022) [14]	To present the current evidence for DPD testing in routine oncological practice.	12	Both prospective and retrospective studies	10,696/NR	NR	HapB3 (322 patients) DPYD*2A (172 patients) D949V (18 patients) DPYD*13 (18 patients)	5-FU, capecitabine or tegafur.	NCI–CTC

5-FU:5-fluorouracil; FIT: fluoropyrimidine-induced toxicity; GI: gastrointestinal; NCI–CTC: National Cancer Institute Common Toxicity Criteria; NR: not reported; PGD: pharmacogenetics-guided dosing; RCTs: randomized control trials.

## Data Availability

Data is contained within the article or Supplementary Material.

## Supplementary Materials

The following supporting information can be downloaded at: https://www.mdpi.com/article/10.3390/ph18050727/s1, Table S1 Complete search strategies for different databases; Table S2. Papers excluded after full-text review and reasons for exclusion; Table S3. Quality analysis of the systematic reviews with/without meta-analysis included according to the AMSTAR-2 tool.
