# Supplementary material for: DPYD Genotyping, Fluoropyrimidine Dosage and Toxicity: An Umbrella Review of Systematic Reviews
Source: Pharmaceuticals (Basel). 2025 May 15;18(5):727. doi: 10.3390/ph18050727 (PMC12115295; doi:10.3390/ph18050727)
Supplement: Supplementary file 1 [file pharmaceuticals-18-00727-s001.zip › pharmaceuticals-3596993-supplementary.pdf]

Table S1. Complete search strategies for different databases

Database	Search Strategy
PubMed	(DPYD[tiab] OR DPD[tiab] OR dihydropyrimidine dehydrogenase[tiab] OR “Dihydropyrimidine Dehydrogenase Deficiency”[MeSH] OR “Dihydrouracil Dehydrogenase (NADP)”[MeSH] OR “5 Fluorouracil”[MeSH] OR Capecitabine[tiab] OR Fluoropyrimidine*[tiab] AND Pharmacogenetics[mesh] OR polymorph*[tiab] OR variant*[tiab] OR mutation*[tiab] OR genotyp*[tiab] OR phenotyp*[tiab])
Scopus	(TITLE-ABS-KEY {pharmacogenetics} OR polymorph* OR variant* OR mutation* OR genotyp* OR phenotyp* AND {dpyd} OR {dpd} OR {dihydropyrimidine dehydrogenase} OR {Dihydropyrimidine Dehydrogenase Deficiency} OR {Dihydrouracil Dehydrogenase} AND {SYSTEMATIC REVIEW})
Web of Science	("DPYD" OR "DPD" OR "dihydropyrimidine dehydrogenase" OR "Dihydropyrimidine Dehydrogenase Deficiency" OR "Dihydrouracil Dehydrogenase (NADP)" OR "5 Fluorouracil" OR "Capecitabine" OR Fluoropyrimidine*) AND ("Pharmacogenetics" OR polymorph* OR variant* OR mutation* OR genotyp* OR phenotyp*) AND "systematic review"
Cochrane Library	#1 MeSH descriptor: [Dihydrouracil Dehydrogenase (NADP)]<br>#2 MeSH descriptor: [Dihydropyrimidine Dehydrogenase Deficiency]<br>#3 MeSH descriptor: [Fluorouracil]<br>#4 MeSH descriptor: [Capecitabine]<br>#5 ("dihydropyrimidine dehydrogenase" OR "DPD" OR "DPYD"),ab,kw<br>#6 #1 OR #2 OR #3 OR #4 OR #5 in Cochrane Reviews<br>#7 MeSH descriptor: [Pharmacogenetics]<br>#8 (polymorph* OR variant* OR mutation* OR genotyp* OR phenotyp*)<br>,ab,kw<br>#9 #7 OR #8 in Cochrane Reviews<br>**#10 #6 AND #9**

Table S2. Papers excluded after full-text review and reasons for exclusion.

Publication	Reason for Exclusion
Pathogenic DPYD Variants and Treatment-Related Mortality in Patients Receiving Fluoropyrimidine Chemotherapy: A Systematic Review and Meta-Analysis	Unable to provide appropriate data
Pharmacogenetics Role of Genetic Variants in Immune-Related Factors: A Systematic Review Focusing on mCRC	Unable to extract genotype
Predicting drug response and toxicity in metastatic colorectal cancer: the role of germline markers	Reviews not following systematic review methodology
Pharmacogenetics: from bench to byte--an update of guidelines	Unable to provide appropriate data
Fluoropyrimidine and platinum toxicity pharmacogenetics: An umbrella review of systematic reviews and meta-analyses,	Umbrella review
Pharmacogenetic testing-guided treatment for oncology: an overview of reviews	Umbrella review
Pharmacogenomics research and clinical implementation in Brazil	Unable to provide appropriate data
Predictive and prognostic biomarkers with therapeutic targets in breast, colorectal, and non-small cell lung cancers: A systemic review of current development, evidence, and recommendation	Reviews not following systematic review methodology
Pharmacogenetics in colorectal cancer: A systematic review	Unable to extract genotype
Advances and challenges in fluoropyrimidine pharmacogenomics and pharmacogenetics	Reviews not following systematic review methodology

Table S3. Quality analysis of the systematic reviews with/without meta-analysis included according to the AMSTAR-2 tool.

**AMSTAR 2 Domains**	**Meulendijks et al. (2015) [15]**	**Terrazzino et al. (2013) [16]**	**Kim et al. (2022) [17]**
1. Did the research questions and inclusion criteria for the review include the components of PICO?	Yes	Yes	Yes
2. Did the report of the review contain an explicit statement that the review methods were established prior to the conduct of the review and did the report justify any significant deviations from the protocol?	No	No	No
3. Did the review authors explain their selection of the study designs for inclusion in the review?	Yes	Yes	Yes
4. Did the review authors use a comprehensive literature search strategy?	Partially Yes	Partially Yes	Yes
5. Did the review authors perform study selection in duplicate?	No	No	Yes
6. Did the review authors perform data extraction in duplicate?	No	Yes	Yes
7. Did the review authors provide a list of excluded studies and justify the exclusions?	No	No	No
8. Did the review authors describe the included studies in adequate detail?	Yes	Yes	Yes
9. Did the review authors use a satisfactory technique for assessing the RoB in individual studies that were included in the review?	Yes	Yes	Yes
10. Did the review authors report on the sources of funding for the studies included in the review?	No	No	No
11. If MA was performed did the review authors use appropriate methods for statistical combination of results?	Yes	Yes	Yes
12. If MA was performed, did the review authors assess the potential impact of RoB in individual studies on the results of the MA or other evidence synthesis?	Yes	Yes	Yes
13. Did the review authors account for RoB in individual studies when interpreting/discussing the results of the review?	Yes	Yes	Yes
14. Did the review authors provide a satisfactory explanation for, and discussion of, any heterogeneity observed in the results of the review?	Yes	Yes	Yes

15. If they performed quantitative synthesis did the review authors carry out an adequate investigation of publication bias (small study bias) and discuss its likely impact on the results of the review?	Yes	Yes	Yes
16. Did the review authors report any potential sources of conflict of interest, including any funding they received for conducting the review?	Yes	Yes	Yes
Confidence level	Critically low	Critically low	Critically low

**AMSTAR 2 Domains**	**Conti et al. (2020) [11]**	**Rosmarin et al. (2014) [10]**	**Glewis et al. (2022) [13]**
1. Did the research questions and inclusion criteria for the review include the components of PICO?	Yes	Yes	Yes
2. Did the report of the review contain an explicit statement that the review methods were established prior to the conduct of the review and did the report justify any significant deviations from the protocol?	No	No	Yes
3. Did the review authors explain their selection of the study designs for inclusion in the review?	Yes	No	Yes
4. Did the review authors use a comprehensive literature search strategy?	Partially Yes	No	Yes
5. Did the review authors perform study selection in duplicate?	Yes	No	Yes
6. Did the review authors perform data extraction in duplicate?	No	Yes	Yes
7. Did the review authors provide a list of excluded studies and justify the exclusions?	No	No	No
8. Did the review authors describe the included studies in adequate detail?	No	Yes	Yes
9. Did the review authors use a satisfactory technique for assessing the RoB in individual studies that were included in the review?	No	No	Yes
10. Did the review authors report on the sources of funding for the studies included in the review?	No	No	No
11. If MA was performed did the review authors use appropriate methods for statistical combination of results?	No MA	Yes	Yes
12. If MA was performed, did the review authors assess the potential impact of RoB in individual studies on the results of the MA or other evidence synthesis?	No MA	No	Yes

13. Did the review authors account for RoB in individual studies when interpreting/discussing the results of the review?	No	No	Yes
14. Did the review authors provide a satisfactory explanation for, and discussion of, any heterogeneity observed in the results of the review?	No	Yes	Yes
15. If they performed quantitative synthesis did the review authors carry out an adequate investigation of publication bias (small study bias) and discuss its likely impact on the results of the review?	No MA	Yes	Yes
16. Did the review authors report any potential sources of conflict of interest, including any funding they received for conducting the review?	Yes	Yes	Yes
Confidence level	Critically low	Critically low	Low

**AMSTAR 2 Domains**	**Paulsen et al. (2022) [14]**	**Ontario Health (2021) [12]**
1. Did the research questions and inclusion criteria for the review include the components of PICO?	Yes	Yes
2. Did the report of the review contain an explicit statement that the review methods were established prior to the conduct of the review and did the report justify any significant deviations from the protocol?	No	Yes
3. Did the review authors explain their selection of the study designs for inclusion in the review?	Yes	Yes
4. Did the review authors use a comprehensive literature search strategy?	Yes	Yes
5. Did the review authors perform study selection in duplicate?	Yes	No
6. Did the review authors perform data extraction in duplicate?	No	No
7. Did the review authors provide a list of excluded studies and justify the exclusions?	Yes	Yes
8. Did the review authors describe the included studies in adequate detail?	Yes	Yes
9. Did the review authors use a satisfactory technique for assessing the RoB in individual studies that were included in the review?	No	Yes
10. Did the review authors report on the sources of funding for the studies included in the review?	No	No

11. If MA was performed did the review authors use appropriate methods for statistical combination of results?	No MA	Yes
12. If MA was performed, did the review authors assess the potential impact of RoB in individual studies on the results of the MA or other evidence synthesis?	No MA	Yes
13. Did the review authors account for RoB in individual studies when interpreting/discussing the results of the review?	No	Yes
14. Did the review authors provide a satisfactory explanation for, and discussion of, any heterogeneity observed in the results of the review?	No	Yes
15. If they performed quantitative synthesis did the review authors carry out an adequate investigation of publication bias (small study bias) and discuss its likely impact on the results of the review?	No MA	Yes
16. Did the review authors report any potential sources of conflict of interest, including any funding they received for conducting the review?	Yes	Yes
Confidence level	Critically low	Moderate

MA: Meta-analysis, PICO: Patients, intervention, comparison and outcomes, RoB: Risk of Bias
